# Identifying risk of death in children hospitalized with community-acquired pneumonia

**DOI:** 10.2471/BLT.22.289000

**Published:** 2023-02-21

**Authors:** Shally Awasthi, Anuj Kumar Pandey, Shambhavi Mishra, on behalf of the CAP Study Group

**Affiliations:** a Department of Pediatrics, King George’s Medical University, Shah Mina Road, Lucknow, Uttar Pradesh-226003, India.

## Abstract

**Objective:**

To externally validate a tool developed by the Pneumonia Research Partnership to Assess WHO Recommendations study group for identification of the risk of death in children hospitalized with community-acquired pneumonia, the PREPARE tool.

**Methods:**

We did a secondary analysis of data collected during hospital-based surveillance of children with community-acquired pneumonia in northern India from January 2015 to February 2022. We included children aged 2–59 months with pulse oximetry assessment. We used multivariable backward stepwise logistic regression analysis to assess the strength of association of the PREPARE variables (except hypothermia) with pneumonia-related death. We estimated sensitivity, specificity, and positive and negative likelihood ratios of the PREPARE score at cut-off scores ≥ 3, ≥ 4 and ≥ 5.

**Findings:**

Of 10 943 children screened, 6745 (61.6%) were included in our analysis, of whom 93 (1.4%) died. Age of < 1 year, female sex, weight-for-age < −3 standard deviations, respiratory rate of ≥ 20 breaths/min higher than the age-specific cut-off, and lethargy, convulsions, cyanosis and blood oxygen saturation < 90% were associated with death. In the validation, the PREPARE score had the highest sensitivity (79.6%) with concurrent highest specificity (72.5%) to identify hospitalized children at risk of death from community-acquired pneumonia at a cut-off score of ≥ 5. Area under curve was 0.82 (95% confidence interval: 0.77–0.86).

**Conclusion:**

The PREPARE tool with pulse oximetry showed good discriminatory ability on external validation in northern India. The tool can be used to assess risk of death of hospitalized children aged 2–59 months with community-acquired pneumonia for early referral to higher-level facilities.

## Résumé

**Objectif:**

Procéder à une validation externe de l'outil PREPARE, un outil développé par le groupe d'étude Pneumonia Research Partnership to Assess WHO Recommendations afin d'identifier le risque de décès chez les enfants hospitalisés pour une pneumonie acquise dans la communauté.

**Méthodes:**

Nous avons mené une analyse secondaire des données récoltées dans le cadre de la surveillance en milieu hospitalier d'enfants souffrant d'une pneumonie acquise dans la communauté dans le nord de l'Inde entre janvier 2015 et février 2022. Nous y avons inclus des enfants âgés de 2 à 59 mois avec oxymétrie de pouls. Nous avons ensuite effectué une analyse multivariée de régression logistique selon la méthode pas à pas descendante en vue d'évaluer la force de l'association des variables PREPARE (à l'exception de l'hypothermie) avec les décès causés par une pneumonie. Enfin, nous avons estimé la sensibilité, la spécificité ainsi que les rapports de vraisemblance positif et négatif du score PREPARE à des seuils ≥ 3, ≥ 4 et ≥ 5.

**Résultats:**

Sur 10 943 enfants examinés, 6745 (61,6%) ont été repris dans notre analyse. Parmi eux, 93 (1,4%) sont décédés. Les critères associés au décès étaient un âge < 1 an, le sexe féminin, un poids pour leur âge < −3 d'écart type, une fréquence respiratoire ≥ 20 respirations/min (plus élevée que le seuil propre à cette catégorie d'âge), ainsi qu'une léthargie, des convulsions, une cyanose et une saturation en oxygène du sang < 90%. Au cours de la validation, le score PREPARE a présenté les niveaux de sensibilité (79,6%) et de spécificité (72,5%) les plus élevés en matière d'identification des enfants hospitalisés présentant un risque de décès dû à une pneumonie acquise dans la communauté à un seuil ≥ 5. L'aire sous la courbe était de 0,82 (intervalle de confiance de 95%: 0,77–0,86).

**Conclusion:**

L'outil PREPARE avec oxymétrie de pouls a démontré une bonne capacité de discernement lors de la validation externe réalisée dans le nord de l'Inde. Cet outil peut servir à évaluer le risque de décès que présentent les enfants hospitalisés pour une pneumonie acquise dans la communauté et âgés de 2 à 59 mois, afin de les transférer rapidement dans des établissements de niveau supérieur.

## Resumen

**Objetivo:**

Validar de manera externa la herramienta PREPARE, una herramienta que ha desarrollado el grupo de estudio Pneumonia Research Partnership to Assess WHO Recommendations para la identificación del riesgo de muerte en niños hospitalizados por neumonía extrahospitalaria.

**Métodos:**

Se realizó un análisis secundario de los datos recopilados durante la vigilancia hospitalaria de niños con neumonía extrahospitalaria en el norte de la India desde enero de 2015 hasta febrero de 2022. Se incluyeron niños de 2 a 59 meses con evaluación de pulsioximetría. Se utilizó un análisis multivariable de regresión logística gradual regresiva para evaluar la fuerza de asociación de las variables PREPARE (excepto la hipotermia) con la muerte relacionada con la neumonía. Se estimaron la sensibilidad, la especificidad y los cocientes de probabilidad positivos y negativos de la puntuación PREPARE en puntuaciones de corte ≥3, ≥4 y ≥5.

**Resultados:**

De 10 943 niños examinados, se incluyeron en nuestro análisis 6 745 (61,6 %), de los que murieron 93 (1,4 %). La edad <1 año, el sexo femenino, el peso para la edad <-3 desviaciones estándar, una frecuencia respiratoria ≥20 respiraciones/min superior al valor de corte específico para la edad, y el letargo, las convulsiones, la cianosis y la saturación de oxígeno en sangre <90 % se asociaron con la muerte. En la validación, la puntuación PREPARE tuvo la mayor sensibilidad (79,6 %) con la mayor especificidad concurrente (72,5 %) para identificar a los niños hospitalizados con riesgo de muerte por neumonía extrahospitalaria con una puntuación de corte ≥5. El área bajo la curva fue de 0,82 (intervalo de confianza del 95 %: 0,77-0,86).

**Conclusión:**

La herramienta PREPARE con pulsioximetría mostró una buena capacidad discriminatoria en la validación externa en el norte de la India. Se puede utilizar para evaluar el riesgo de muerte de niños hospitalizados de entre 2 y 59 meses de edad con neumonía extrahospitalaria para su derivación anticipada a centros de nivel superior.

## ملخص

**الغرض:**

التحقق بواسطة جهة خارجية من أداة ابتكرتها مجموعة دراسة ضمن شراكة أبحاث الالتهاب الرئوي لتقييم توصيات منظمة الصحة العالمية للتعرف على خطر الوفاة على الأطفال المقيمين في المستشفى بسبب التهاب رئوي بعدوى من المحيطين في المجتمع، وهي أداة PREPARE.

**الطريقة:**

أجرينا تحليلاً ثانويًا للبيانات التي تم جمعها في أثناء المتابعة في المستشفى للأطفال المصابين بالتهاب رئوي بسبب عدوى من المحيطين في المجتمع في شمال الهند بدءًا من شهر يناير/كانون أول 2015 إلى شهر فبراير/شباط 2022. أدرجنا الأطفال في عمر 2 إلى 59 شهرًا ممن لديهم تقييم لقياس التأكسج النبضي. استخدمنا تحليل الانحدار اللوجستي التدريجي الخلفي متعدد المتغيرات لتقييم قوة ارتباط متغيرات أداة PREPARE (باستثناء انخفاض درجة الحرارة) بالوفاة بسبب الالتهاب الرئوي. وضعنا تقديرًا للحساسية والتحديد والمعدلات الموجبة والسالبة لاحتمالات درجة أداة PREPARE عند الدرجات الفاصلة ≥ 3 و≥ 4 و≥ 5.

**النتائج:**

من بين 10943 طفلاً جرى فحصهم، تم إدراج 6745 (%61.6) في تحليلنا، ومن بينهم 93 (%1.4) لاقوا حتفهم. العمر الأقل من عام واحد، والإناث، والانحراف المعياري الأقل من -3 للوزن بالنسبة إلى العمر، ومعدل التنفس الذي يزيد بمقدار ≥ 20 نفس/دقيقة عن حد الفصل المستند إلى العمر، والنعاس، والتشنجات، والزراق، والتشبع بالأكسجين الأقل من %90 كانت العوامل المرتبطة بالوفاة. في سياق التحقق، وصلت درجة أداة PREPARE إلى أعلى حساسية (%79.6) مع أعلى تحديد متزامن (%72.5) للتعرف على الأطفال المقيمين في المستشفى المعرضين لخطر الوفاة بسبب التهاب رئوي بعدوى من المحيطين في المجتمع عند درجة فاصلة تبلغ ≥ 5. كانت المنطقة تحت المنحنى تبلغ 0.82 (بفاصل ثقة مقداره %95: 0.77 إلى 0.86).

**الاستنتاج:**

أظهرت أداة PREPARE مع قياس التأكسج النبضي قدرة تمييز جيدة في التحقق من جانب جهة خارجية في شمال الهند. يمكن استخدام الأداة لتقييم خطر وفاة الأطفال المقيمين في المستشفى في عمر 2 إلى 59 شهرًا من المصابين بالتهاب رئوي بعدوى من المحيطين في المجتمع من أجل إحالتهم مبكرًا إلى منشآت أعلى في المستوى.

## 摘要

**目的:**

旨在从外部验证肺炎研究伙伴关系开发的工具，即用于评估世卫组织建议研究小组确定社区获得性肺炎住院儿童的死亡风险情况的 PREPARE 工具。

**方法:**

我们对 2015 年 1 月至 2022 年 2 月在印度北部地区对社区获得性肺炎患儿进行医院监测期间收集的数据进行了二次分析。我们纳入了进行脉搏血氧饱和度评估的年龄在 2-59 个月的儿童。我们使用多变量逻辑向后逐步回归分析来评估 PREPARE 变量（体温过低除外）与肺炎相关死亡的相关性强度。我们估计了 PREPARE 评分在临界值 ≥ 3、≥ 4 和 ≥ 5 时的敏感性、特异性以及阳性和阴性似然比。

**结果:**

在筛查的 10,943 名儿童中，6,745 名 (61.6%) 被纳入我们的分析，其中 93 名 (1.4%) 死亡。年龄 < 1 岁，女性，年龄别体重 < -3 个标准差，呼吸频率比特定年龄临界值高 ≥ 20 次/分，嗜睡、抽搐、发绀和血氧饱和度 < 90% 与死亡相关。在验证中，当临界值 ≥ 5 时，PREPARE 评分在确定社区获得性肺炎住院儿童死亡风险方面同时具有最高的敏感性 (79.6%) 和最高的特异性 (72.5%)。曲线下面积为 0.82（95% 置信区间：0.77–0.86）。

**结论:**

在印度北部地区，采用脉搏血氧饱和度评估法的 PREPARE 工具在外部验证方面表现出了良好的判别能力。该工具可用于评估年龄在 2-59 个月的社区获得性肺炎住院儿童的死亡风险情况，以便尽早转诊到更高级别的机构。

## Резюме

**Цель:**

Провести внешнюю валидацию инструмента PREPARE, разработанного исследовательской группой Pneumonia Research Partnership to Assess WHO Recommendations для определения риска смерти среди детей, госпитализированных с внебольничной пневмонией.

**Методы:**

С января 2015 года по февраль 2022 года на севере Индии был проведен дополнительный анализ данных, собранных в ходе стационарного наблюдения за детьми с внебольничной пневмонией. В него были включены дети в возрасте 2–59 месяцев, для которых имелись данные пульсоксиметрии. Для оценки степени сходства переменных PREPARE (за исключением гипотермии) со смертью от пневмонии использовали многомерный обратный пошаговый логистический регрессионный анализ. Использовали такие показатели, как чувствительность, специфичность, положительное и отрицательное отношение правдоподобия, для оценки по шкале PREPARE при минимально допустимых баллах ≥ 3, ≥ 4 и ≥ 5.

**Результаты:**

Из 10 943 детей, прошедших скрининг, 6745 (61,6%) были включены в анализ, из них 93 (1,4%) скончались. Со смертью были связаны следующие факторы: возраст до 1 года, женский пол, вес для данного возраста < –3 стандартных отклонений, частота дыхания, превышающая возрастную норму на 20 вдохов/мин и более, а также вялость, судороги, цианоз и насыщение крови кислородом < 90%. В ходе валидации оценка по шкале PREPARE продемонстрировала самую высокую чувствительность (79,6%) одновременно при самой высокой специфичности (72,5%) для выявления госпитализированных детей с риском смерти от внебольничной пневмонии при минимально допустимом балле ≥ 5. Площадь под кривой составила 0,82 (95%-й ДИ: 0,77–0,86).

**Вывод:**

При использовании пульсоксиметрии инструмент PREPARE продемонстрировал хорошую дискриминационную способность в ходе внешней валидации в Северной Индии. Этот инструмент может использоваться для оценки риска смерти госпитализированных детей в возрасте 2–59 месяцев с внебольничной пневмонией для раннего направления в учреждения более высокого уровня.

## Introduction

Community-acquired pneumonia is the leading cause of death in children younger than 5 years, accounting for 14.2% (0.74/5.2 million) of deaths in this age group worldwide in 2019.^1^^,^^2^ In 2018, 0.8 million children globally died from community-acquired pneumonia, of which 0.13 million were in India, that is about 14 deaths every hour.^3^ The World Health Organization (WHO) has classified community-acquired pneumonia as: (i) pneumonia, where there is fast breathing with or without chest indrawing; and (ii) severe pneumonia, where one or more danger signs are present, such as inability to drink, persistent vomiting, convulsions, lethargy or unconsciousness, stridor in a calm child or severe malnutrition.^4^ Most deaths related to community-acquired pneumonia occur in cases of severe pneumonia.^5^ Therefore, patients with severe community-acquired pneumonia must be hospitalized for optimal care, ideally in a tertiary-care facility. Generally, families opt for a facility closest to their home for treatment of illness.^6^ This choice increases the risk of hospital mortality. Therefore, in 2022, the Pneumonia Research Partnership to Assess WHO Recommendations (PREPARE) study group developed a tool to help identify the risk of hospital deaths in patients with severe community-acquired pneumonia (the PREPARE tool).^7^ In developing this tool, data from 27 388 children aged 2–59 months from low- and middle-income countries were used. The tool has two scores, one with pulse oximetry and another without pulse oximetry. Both scores have good sensitivity and specificity to identify children at risk of death at specific cut-off points, and good discriminatory ability.^7^ The tool uses data that are routinely collected in most hospitals. The PREPARE tool is the latest of several tools developed to identify patients with community-acquired pneumonia at risk of death.^8^^–^^11^ However, before the widespread use of the PREPARE tool is promoted, external validation needs to be done. Therefore, the objective of our study was to externally validate this tool with pulse oximetry for identification of children aged 2–59 months with community-acquired pneumonia at risk of death.

## Method

### Study design

We used data collected in a prospective, multisite ongoing hospital-based surveillance system on community-acquired pneumonia in children aged 2–59 months. The surveillance included four districts, namely Lucknow and Etawah in Uttar Pradesh, and Patna and Darbhanga in Bihar, India, and started on 1 January 2015.^12^ The system included children hospitalized for community-acquired pneumonia in public and private hospitals. We applied the PREPARE tool retrospectively to these data to assess its ability to predict hospital mortality related to pneumonia. The surveillance system had collected all the data used by the tool except for two variables: lower chest indrawing, and body temperature measured by thermometer. Therefore, for our conservative external validation, we did not include these two variables in determining the PREPARE score. We used the Transparent Reporting of a Multivariable Prediction Model for Individual Prognosis or Diagnosis checklist for the external validation of the PREPARE tool.^13^

### Sample size

For an expected sensitivity and specificity of 70%^7^ of the PREPARE score on external validation, with 10% absolute precision and 95% confidence interval (CI) with a 1.4% prevalence of hospital mortality, the minimum required sample was 5763.^14^

### Data source

We collected data on children hospitalized with community-acquired pneumonia aged 2–59 months from January 2015 to February 2022 from the hospitals-based network in the selected districts. In the original surveillance, trained surveillance officers recruited children with community-acquired pneumonia from the network hospitals after parental consent. Inclusion criteria were: (i) 2–59 months of age; (ii) hospitalization with symptoms of community-acquired pneumonia as defined by WHO; (iii) resident of the project district; (iv) illness of < 14 days; (v) no previous hospitalization for community-acquired pneumonia nor recruitment in the surveillance; and (vi) parental consent to participate. We excluded children without pulse oximetry data from our analysis.

### Variables

The sociodemographic and clinical variables included were age, sex, weight-for-age, immunization status, respiratory rate, lethargy and/or unconsciousness, comorbidities, convulsions, cyanosis and blood oxygen saturation. Our outcome measure was hospital death from community-acquired pneumonia.

Comorbidities included congenital heart disease and a history of fast breathing, and having a cough three times or more in 6 months as a surrogate marker of asthma. We defined tachypnoea as (greater than/equal to) ≥ 50 breaths/min for children aged 2–11 months and (greater than/equal to) ≥ 40 breaths/min for children aged 12–59 months.^4^^,^^15^ We categorized nutrition status of the children as: severe malnutrition with weight-for-age z scores < −3 standard deviation (SD); moderate malnutrition with weight-for-age z scores −3 SD to −2 SD; and adequate nutrition as weight-for-age z scores > −2 SD.^16^ We categorized blood oxygen saturation of < 90% as severe hypoxemia, 90–92% as mild hypoxemia and 93–100% as normal blood oxygen. All variables included in our analysis had been recorded at initial enrolment in the surveillance system. All deaths included in our analysis occurred during the hospital stay for this episode of community-acquired pneumonia.

We categorized children as fully immunized if they had received all vaccines as per the national immunization schedule of India in their first year of life.^17^ Because pneumococcal conjugate vaccine was rolled out in a phased manner from 1 June 2017 onwards but staggered during the coronavirus disease 2019 (COVID-19) pandemic, we did not include this vaccine in the definition of fully vaccinated.^18^^,^^19^

The PREPARE tool has one score that includes hypoxia, measured by pulse oximetry, which ranges from 0 to 17 and another score that does not include hypoxia, which ranges from 0 to 20.^7^ The second score allows resource-constrained areas with no access to pulse oximetry and skilled human resources to also use the PREPARE tool. The primary data from the surveillance system did not include the presence of lower chest indrawing as this symptom was not used in the revised WHO classification of community-acquired pneumonia.^5^ Since lack of this information does not affect the coding in the PREPARE score with pulse oximetry, we only included children whose pulse oximetry had been measured. As we did not have data on body temperature, our PREPARE risk assessment score ranged from 0 to 14 (three points for the presence of hypothermia were not included). We excluded children who had data missing on any of the variables we used in our scoring.

### Data analysis

We compared sociodemographic and clinical variables of hospitalized children who died of community-acquired pneumonia and those who survived the episode. We used numbers and percentages for categorical variables. We converted continuous variables into categorical variables based on recommended thresholds before developing our model to facilitate external validation of the PREPARE tool.

We used multivariable backward stepwise logistic regression analysis to identify risk factors for hospital mortality for community-acquired pneumonia. We included variables with a *P*-value ≤ 0.1 in the univariable analysis in the regression analysis. We calculated adjusted odds ratios (aOR) and 95% CIs of the association of sociodemographic and clinical variables with hospital mortality. This model also assessed the comparability of our data with the PREPARE model with pulse oximetry. We considered a two-tailed *P*-value of < 0.05 as statistically significant.

We used cut-off points of ≥ 3, ≥ 4 and ≥ 5 in PREPARE scores in our sensitivity and specificity analysis of the PREPARE tool, as reported in the development and internal validation of the tool.^7^ We calculated positive and negative likelihood ratios with 95% CI and the Youden index to assess the improvement in likelihood of correctly identifying hospital mortality. We constructed receiver operating characteristic curves for the PREPARE tool with pulse oximetry. We used area under the curve with 95% CI to assess the discriminatory ability of score. The scale used to qualify the discriminatory ability of score was area under the curve ≥ 0.90 for excellent discrimination, area under the curve 0.80 to 0.89 for good discrimination, area under the curve 0.70 to 0.79 for fair discrimination and area under the curve < 0.70 for poor discrimination.^20^^,^^21^ We used SPSS version 24 (SPSS Inc., Chicago, United States of America) for all statistical analyses.

### Ethical considerations

The primary surveillance study was approved by the Ethics Review Committee of each participating site: (i) King George`s Medical University, Lucknow; (ii) Darbhanga Medical College & Hospital, Darbhanga; (iii) Patna Medical College and Hospital, Patna; and (iv) UP Rural Institute of Medical Sciences & Research, Etawah. The caregivers or guardians of children signed a written, informed consent form for participation in the surveillance and we used anonymized data for the external validation of the PREPARE tool.

## Results

From January 2015 to February 2022, 10 943 children were screened in the four districts. We excluded 131 children with no parental consent and 4067 children without pulse oximetry measurements. Thus, we included 6745 (61.6%) children with hospitalized with community-acquired pneumonia in the analysis. Of these children, 1.4% (93/6745) died in hospital, which is similar to those without pulse-oximetry, also 1.4% (55/4067). The mean PREPARE scores were 3.36 (95% CI: 3.31–3.41) for children who survived and 6.27 (95% CI: 5.76–6.78) for children who died.

The percentages of children who were fully vaccinated were similar across the age groups: 79.0% (2258/2859) of children aged 2–5 months, 82.1% (1519/1850) of children aged 6–11 months and 81.9% (1667/2036) of children aged 12–59 months. Overall, 80.7% (5444/6745) of the children were fully immunized.

Comparison of sociodemographic and clinical variables of hospitalized children who survived and who died is given in Table 1. Significant differences in mortality were found with all variables (all *P* < 0.001), except age group.

**Table 1:** Sociodemographic and clinical characteristics of children hospitalized with community-acquired pneumonia, by outcome, northern India, 2015–2022

Characteristic	No. (%)		*P*
	Survived (*n* = 6652)	Died (*n* = 93)	
**Age group, in months**			0.17
12–59	2016 (30.3)	20 (21.5)	
6–11	1823 (27.4)	27 (29.0)	
2–5	2813 (42.3)	46 (49.5)	
**Sex **			< 0.001
Male	4708 (70.8)	49 (52.7)	
Female	1944 (29.2)	44 (47.3)	
**Comorbidities **			< 0.001
No	6479 (97.4)	77 (82.8)	
Yes	173 (2.6)	16 (17.2)	
**Immunization status^a^**			< 0.001
Complete for age	5386 (81.0)	58 (62.4)	
Incomplete for age or unimmunized	1266 (19.0)	35 (37.6)	
**Weight-for-age z score**			< 0.001
≥ −2 SD	4302 (64.7)	34 (36.6)	
−3 to −2 SD	1306 (19.6)	20 (21.5)	
< −3 SD	1044 (15.7)	39 (41.9)	
**Respiratory rate, breaths/min**			< 0.001
≤ age-specific cut-off	1433 (21.5)	7 (7.5)	
0–9 more than age-specific cut-off	1999 (30.1)	18 (19.4)	
10–19 more than age-specific cut-off	2164 (32.5)	23 (24.7)	
≥ 20 more than age-specific cut-off	1056 (15.9)	45 (48.4)	
**Lethargy and/or unconsciousness**			< 0.001
No	3203 (48.2)	27 (29.0)	
Yes	3449 (51.8)	66 (71.0)	
**Convulsions**			< 0.001
No	6297 (94.7)	70 (75.3)	
Yes	355 (5.3)	23 (24.7)	
**Cyanosis **			< 0.001
No	6591 (99.1)	85 (91.4)	
Yes	61 (0.9)	8 (8.6)	
**Oxygen saturation, %**			< 0.001
< 90	935 (14.1)	45 (48.4)	
90–92	1275 (19.2)	19 (20.4)	
93–100	4442 (66.8)	29 (31.2)	

SD: standard deviation.

^a^ Excluding pneumococcal conjugate vaccine.

Multivariable backward stepwise logistic regression analysis of sociodemographic and clinical variables associated with hospital mortality is shown in Table 2. Female sex, weight-for-age < −3 SD, respiratory rate ≥ 20 breaths more than the age-specific cut-off, lethargy and/or unconsciousness, convulsions, cyanosis and blood oxygen saturation < 90% were all significantly associated with death.

**Table 2:** Logistic regression analysis of sociodemographic and clinical variables associated with death in children hospitalized with community-acquired pneumonia, northern India, 2015–2022

Variable	aOR (95% CI)
**Age group, in months**	
12–59	Reference
6–11	2.28 (1.22–4.25)
2–5	1.77 (1.01–3.11)
**Sex (female)**	2.42 (1.56–3.75)
**Weight-for-age z score**	
≥ −2 SD	Reference
−3 to −2 SD	1.42 (0.79–2.55)
< −3 SD	3.22 (1.96–5.28)
**Respiratory rate (breaths/min) **	
≤ age-specific cut-off	Reference
0–9 more than age-specific cut-off	1.68 (0.69–4.10)
10–19 more than age-specific cut-off	1.48 (0.62–3.54)
≥ 20 more than age-specific cut-off	4.79 (2.06–11.16)
**Comorbidities**	5.05 (2.73–9.32)
**Lethargy and/or unconsciousness**	1.79 (1.11–2.90)
**Convulsion**	3.37 (1.95–5.81)
**Cyanosis**	3.38 (1.35–8.46)
**Oxygen saturation category, %**	
< 90	3.82 (2.29–6.36)
90–92	1.71 (0.93–3.12)
93–100	Reference

aOR: adjusted odds ratio; CI: confidence interval; SD: standard deviation.

**Fig. 1:**
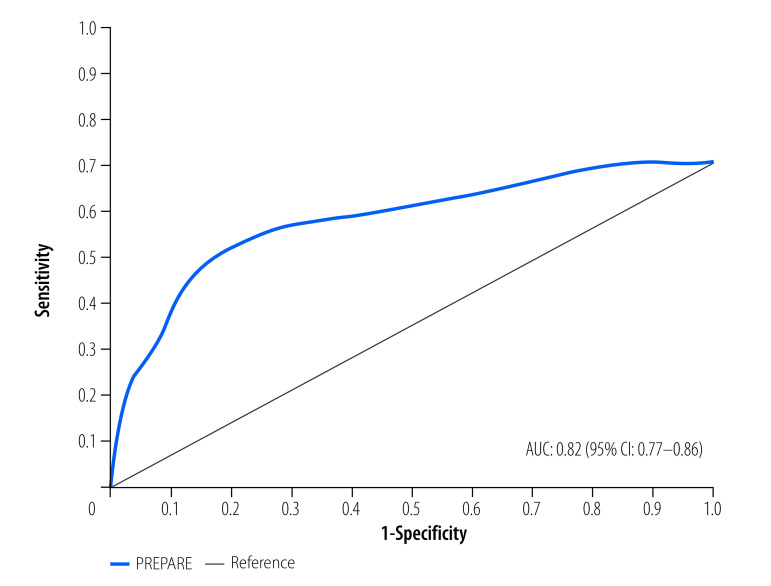
Receiver operating characteristic curve for PREPARE scores to identify the risk of death in children aged 2–59 months hospitalized with community-acquired pneumonia

AUC: area under the curve; CI: confidence interval; PREPARE: Pneumonia Research Partnership to Assess WHO Recommendations.

The PREPARE tool had the highest concurrent sensitivity and specificity to identify hospital mortality at a cut-off score of ≥ 5: 79.6% sensitivity and 72.5% specificity with a positive likelihood ratio of 2.90 (95% CI: 2.59–3.23) and negative likelihood ratio of 0.28 (95% CI: 0.19–0.42; Table 3). The Youden index showed moderate discriminatory power (0.52) at a score of ≥ 5. The overall area under the curve of the PREPARE risk score was 0.82 (95% CI: 0.77–0.86; Fig. 1). The area under the curve of the receiver operating characteristic curve using mortality and PREPARE score at a cut-off of ≥ 5 was 0.71 (95% CI: 0.65–0.77) indicating fair discriminatory power.

**Table 3:** Sensitivity and specificity of the PREPARE tool with pulse oximetry to identify hospitalized children with community-acquired pneumonia at risk of death, by cut-off score

PREPARE score cut-off	Sensitivity, %	Specificity, %	Positive likelihood ratio (95% CI)	Negative likelihood ratio (95% CI)	Youden index
≥ 3	90.3	38.8	1.48 (1.38–1.58)	0.25 (0.13–0.46)	0.29
≥ 4	83.9	57.5	1.97 (1.80–2.16)	0.28 (0.18–0.45)	0.41
≥ 5	79.6	72.5	2.90 (2.59–3.23)	0.28 (0.19–0.42)	0.52

CI: Confidence interval; PREPARE: Pneumonia Research Partnership to Assess WHO Recommendations.

Of the 93 children who died in hospital of community-acquired pneumonia, hospital mortality was 90.3% (84/93) at a cut-off score ≥ 3, 83.9% (78/93) at ≥ 4 and 79.6% (74/93) at ≥ 5. When the PREPARE score cut-off of < 5 was used, the risk of hospital mortality was misclassified in 19 children who died (20.4% of the 93 deaths). Children who had been misclassified were aged between 6 and 11 months (9/19, 47.4%), had a weight-for-age z score of ≥ −2 SD (16/19, 84.2%), a respiratory rate of 10–19 breaths/min higher than the age-specific cut-off (8/19, 42.1%), had no convulsions (19/19, 100.0%), and had an oxygen saturation level of > 93% (10/19, 52.6%).

Comparison of the PREPARE tool to other risk assessment tools – such as the Respiratory Index of Severity in Children (RISC) from South Africa,^8^ the Modified Respiratory Index of Severity in Children (mRISC) from Kenya,^9^ RISC-Malawi from Malawi,^10^ and Pneumonia Etiology Research for Child Health (PERCH) from Bangladesh, Gambia, Kenya, Mali, South Africa, Thailand and Zambia^11^ – that have been developed to identify patients with pneumonia at risk of death during hospital stay is shown in Table 4. Even though the tools have different predictor variables, weight-for-age z-score is used in all of them. Oxygen saturation is included in all except mRISC, unconsciousness is included in all except the PERCH tool, and sex is not included in the RISC and mRISC tools. All of these variables are included in the PREPARE tool. The RISC score had the highest area under the curve. However, this score is based on children aged 0–24 months whereas community-acquired pneumonia affects children up to 5 years.^1^ As such, the RISC tool has limited use. The PREPARE tool was developed for children aged 2–59 months using data from 20 countries which makes it more generalizable.

**Table 4:** Variables included in scoring tools developed to estimate risk of death in children hospitalized with community-acquired pneumonia

Variable^a^	Variable included, yes (score assigned) or no					
	RISC (0–24 months)^8^	mRISC (0–59 months)^9^	RISC-Malawi (2–59 months)^10^	PERCH (1–59 months)^11^	PREPARE with pulse oximetry (2–59 months)^7^	PREPARE without pulse oximetry (2–59 months)^7^
**Age, in months**						
2–5	No	No	No	Yes (2)	Yes (2)	Yes (2)
6–11	No	No	No	Yes (2)	Yes (1)	Yes (1)
12–59	No	No	No	Yes (0)	Yes (0)	Yes (0)
**Body temperature, °C**						
< 35.5	No	No	No	No	Yes (3)	Yes (3)
35.5–37.9	No	No	No	No	Yes (0)	Yes (0)
≥ 38	No	No	No	No	Yes (0)	Yes (0)
**Convulsions**	No	No	No	No	Yes (2)	Yes (2)
**Cough**	No	No	No	Yes (–1)	No	No
**Cyanosis**	No	No	No	No	Yes (2)	Yes (3)
**Dehydration**	No	Yes (1)	No	No	No	No
**Sex**						
Male	No	No	Yes (0)	Yes (0)	Yes (0)	Yes (0)
Female	No	No	Yes (1)	Yes (1)	Yes (1)	Yes (1)
**Grunting**	No	No	No	Yes (2)	No	No
**History of night sweats**	No	Yes (–1)	No	No	No	No
**Lower chest indrawing**	Yes (2)	Yes (1)	No	No	Yes (0)	Yes (1)
**Malaria**	No	Yes (–1)	No	No	No	No
**Malaria and chest indrawing**	No	Yes (1)	No	No	No	No
**Duration of illness, in days**						
3–5	No	No	No	Yes (2)	No	No
> 5	No	No	No	Yes (2)	No	No
**Not alert or awake**	No	Yes (2)	No	No	No	No
**Oxygen saturation, %**						
< 90	Yes (3)	No	Yes (5)	Yes (2)	Yes (2)	No
90–92	Yes (0)	No	Yes (1)	Yes (2)	Yes (0)	No
93–100	Yes (0)	No	Yes (0)	Yes (0)	Yes (0)	No
**Refusal to feed**	Yes (1)	Yes (1)	No	No	No	No
**Respiratory rate (breaths/min)**						
≤ Age-specific cut-off	No	No	No	No	Yes (0)	Yes (0)
0–9 higher than age-specific cut-off	No	No	No	No	Yes (0)	Yes (0)
10–19 higher than age-specific cut-off	No	No	No	No	Yes (0)	Yes (0)
≥ 20 higher than age-specific cut-off	No	No	No	No	Yes (1)	Yes (2)
**Unconscious or decreased consciousness**	No	Yes (1)	Yes (5)	No	Yes (1)	Yes (2)
**Unresponsive deep breathing**						
Unresponsive without deep breathing	No	Yes (2)	No	No	No	No
Unresponsive with deep breathing	No	Yes (5)	No	No	No	No
**Wheezing**	Yes (–2)	No	Yes (–1)	No	No	No
**WHO weight-for-age z score**						
≥ −2 SD	Yes (0)	Yes (0)	Yes (0)	Yes (0)	Yes (0)	Yes (0)
−2 to −3 SD	Yes (1)	Yes (1)	Yes (3)	Yes (2)	Yes (2)	Yes (2)
< −3 SD	Yes (2)	Yes (1)	Yes (6)	Yes (3)	Yes (3)	Yes (4)
**Total score at development**	6	8	17	17	17	20
**Sensitivity at development**	94.1% at cut-off score of 3	80.7% at cut-off score of 1	82.0% at cut-off score of 5	74.5% at cut-off score of 5	72.6% at cut-off score of 5	NA
**Specificity at development**	73.6% at cut-off score of 3	72.9% at cut-off score of 1	73.0% at cut-off score of 5	82.3% at cut-off score of 5	76.5% at cut-off score of 5	NA
**Area under the curve at development**	0.92	0.85	0.80	0.76	0.83	0.81
**Year of publication**	2012	2014	2016	2020	2022	2022
**Sample size**	2679	3974	14 665	1994	27 388	27 388
**Region or country**	South Africa	Western Kenya	Malawi	Low- and middle-income countries	Multicountry	Multicountry

mRISC: Modified Respiratory Index of Severity in Children; NA: data not available; PERCH: Pneumonia Etiology Research for Child Health; PREPARE: Pneumonia Research Partnership to Assess WHO Recommendations; RISC: Respiratory Index of Severity in Children; SD: standard deviation; WHO: World Health Organization.

^a^ Applicable to children negative for human immunodeficiency virus.

## Discussion

Our external validation showed that the PREPARE tool with pulse oximetry had good discriminatory ability and the sensitivity and specificity of the tool was highest at a cut-off value of ≥ 5.

Internal validation of the PREPARE score reported an area under the curve of 0.83 (95% CI: 0.81–0.84) for hospital mortality,^7^ which is similar to our findings. The PREPARE tool was also reported to have maximum concurrent sensitivity and specificity at a cut-off of ≥ 5 (72.6% sensitivity and 76.5% specificity) with positive likelihood ratio of 3.09 (95% CI: 2.89–3.30) and negative likelihood ratio of 0.36 (95% CI: 0.31–0.42),^7^ which are comparable to our study. Hence our external validation of the PREPARE tool showed comparable discriminatory capacity. 

In our external validation, the odds of death for all included variables except age were similar to the reported internal validation of the PREPARE tool.^7^ Children aged 2–5 months had statistically higher odds of death on internal validation of the PREPARE tool.^7^ We did not have adequate sample sizes to analyse the mortality differences between the age groups.

Immunization is an important intervention for the prevention of community-acquired pneumonia in children.^22^ The most common etiological agents are *Haemophilus influenzae* and *Streptococcus pneumoniae*.^23^ The *H. influenzae* vaccine was introduced as a part of pentavalent vaccine in India’s national immunization schedule in 2011 in phases. The phased roll-out of the pneumococcal conjugate vaccine started in 2017, so some of the data used in our study were collected before the introduction of this vaccine. The data used in the development of the PREPARE tool were collected before the introduction of the *H. influenzae* and pneumococcal conjugate vaccines in some of the 20 countries from which data were obtained. Therefore, immunization status is not included in the PREPARE tool, although we found immunization to be associated with hospital mortality of community-acquired pneumonia. However, often parents do not have immunization records with them when their child is admitted to hospital, and hence exclusion of immunization seems justified.

Body temperature is a risk factor included in the PREPARE tool as a strong association between hospital mortality and hypothermia has been shown.^7^ Hypothermia has been reported to be associated with sepsis and excess mortality.^24^ Body temperature was not recorded in our data set and hence we could not include it in our external validation of the PREPARE tool. However, even without the inclusion of body temperature, the PREPARE tool showed good test characteristics in our validation, similar to the internal validation.^7^ Therefore, the PREPARE tool with pulse oximetry appears to be an effective tool without data on body temperature.

Chest indrawing was found to have no association with mortality in the PREPARE tool with pulse oximetry and was not assessed in our study. This symptom is included in the RISC and mRISC tools but not in the PERCH and RISC Malawi tools for pneumonia-related mortality in hospitalized patients. The classification and management of community-acquired pneumonia was revised in the 2014 WHO guidelines and chest indrawing was not included. In younger children chest indrawing is a less specific finding because they have more compliant chest walls. However, chest indrawing along with signs of severe respiratory distress calls for attention and intervention.^25^ Therefore, in the PREPARE tool without pulse oximetry, which we did not externally validate, the presence of lower chest indrawing is given a score of +1.

We applied the PREPARE tool using data with pulse oximetry. Hypoxia alone has been reported as a single risk factor for death from pneumonia in children.^26^ We also found that the aOR of mortality in children with hypoxia (blood oxygen saturation < 90%) was 4.02 (95% CI: 2.43–6.65). A systematic review and meta-analysis supported routine evaluation of blood oxygen saturation to identify children with acute lower respiratory infection at increased risk of death.^27^ In line with this recommendation, hypoxemia is incorporated in tools such as RISC, RISC-Malawi and PERCH. Since the COVID-19 pandemic, many health facilities, including peripheral health centres, are using pulse oximeters. We recommend the use of pulse oximetry to document hypoxemia in patients with community-acquired pneumonia.^28^

Our study has some limitations. The data used in our study started to be collected in 2015, before the PREPARE tool was developed. Hence, not all variables included in the original PREPARE tool were available in our data set, specifically body temperature and chest indrawing. Hypothermia has been strongly associated with mortality in the PREPARE internal validation study and hence needs further external validation. As mentioned earlier, chest indrawing lacked association with mortality in the internal validation of the PREPARE tool with pulse oximetry. Some of the data in our study were collected before the introduction of pneumococcal conjugate vaccine and hence mortality may differ in populations with different vaccination coverage. Since our data were collected for another study and several sites were involved, inaccuracies in data collection could have led to misclassification bias of the variables used in the PREPARE tool. We did not validate the PREPARE tool without pulse oximetry as we did not have the necessary sample size for 70% sensitivity and specificity as found in the internal validation of the PREPARE tool.^7^ We used data from northern India only for our validation. Validation of the PREPARE tool in different parts of India and other regions is needed before incorporating it in standard of care guidelines. Similarly, external validation of the PREPARE tool without pulse oximetry should be done.

To conclude, our external validation of the PREPARE tool with pulse oximetry demonstrated that this tool had good discriminatory value for identifying children aged 2–59 months hospitalized with community-acquired pneumonia who are at risk of death. This tool can therefore be used to assess the risk of death in such children for early referral to higher level health-care facilities.
